# Bio-Pathological Functions of Posttranslational Modifications of Histological Biomarkers in Breast Cancer

**DOI:** 10.3390/molecules29174156

**Published:** 2024-09-02

**Authors:** Anca-Narcisa Neagu, Claudiu-Laurentiu Josan, Taniya M. Jayaweera, Hailey Morrissiey, Kaya R. Johnson, Costel C. Darie

**Affiliations:** 1 Laboratory of Animal Histology, Faculty of Biology, “Alexandru Ioan Cuza” University of Iași, Carol I bvd. 20A, 700505 Iasi, Romania; claudiujosan22@gmail.com; 2 Biochemistry & Proteomics Laboratories, Department of Chemistry and Biomolecular Science, Clarkson University, Potsdam, NY 13699-5810, USA; jayawetm@clarkson.edu (T.M.J.); morrisha@clarkson.edu (H.M.); johnsokr@clarkson.edu (K.R.J.)

**Keywords:** biomarkers, posttranslational modification of proteins (PTMs), breast cancer (BC)

## Abstract

Proteins are the most common types of biomarkers used in breast cancer (BC) theranostics and management. By definition, a biomarker must be a relevant, objective, stable, and quantifiable biomolecule or other parameter, but proteins are known to exhibit the most variate and profound structural and functional variation. Thus, the proteome is highly dynamic and permanently reshaped and readapted, according to changing microenvironments, to maintain the local cell and tissue homeostasis. It is known that protein posttranslational modifications (PTMs) can affect all aspects of protein function. In this review, we focused our analysis on the different types of PTMs of histological biomarkers in BC. Thus, we analyzed the most common PTMs, including phosphorylation, acetylation, methylation, ubiquitination, SUMOylation, neddylation, palmitoylation, myristoylation, and glycosylation/sialylation/fucosylation of transcription factors, proliferation marker Ki-67, plasma membrane proteins, and histone modifications. Most of these PTMs occur in the presence of cellular stress. We emphasized that these PTMs interfere with these biomarkers maintenance, turnover and lifespan, nuclear or subcellular localization, structure and function, stabilization or inactivation, initiation or silencing of genomic and non-genomic pathways, including transcriptional activities or signaling pathways, mitosis, proteostasis, cell–cell and cell–extracellular matrix (ECM) interactions, membrane trafficking, and PPIs. Moreover, PTMs of these biomarkers orchestrate all hallmark pathways that are dysregulated in BC, playing both pro- and/or antitumoral and context-specific roles in DNA damage, repair and genomic stability, inactivation/activation of tumor-suppressor genes and oncogenes, phenotypic plasticity, epigenetic regulation of gene expression and non-mutational reprogramming, proliferative signaling, endocytosis, cell death, dysregulated TME, invasion and metastasis, including epithelial–mesenchymal/mesenchymal–epithelial transition (EMT/MET), and resistance to therapy or reversal of multidrug therapy resistance. PTMs occur in the nucleus but also at the plasma membrane and cytoplasmic level and induce biomarker translocation with opposite effects. Analysis of protein PTMs allows for the discovery and validation of new biomarkers in BC, mainly for early diagnosis, like extracellular vesicle glycosylation, which may be considered as a potential source of circulating cancer biomarkers.

## 1. Introduction

In bio-medical research and clinical practice, a biomarker is defined as a relevant, objective, quantifiable and statistically validated bio-medical feature/characteristic/information/sign/process/propriety/biomolecule/product that can be accurately and repeatably measured to indicate/evaluate/assess/monitor normal physiological processes, pathogenic mechanisms or pharmacological responses to therapy [1,2]. In the oncological domain, due to the high intra- and inter-heterogeneity of tumors, combinations of biomarkers are preferred and required in BC diagnosis and treatment [3]. Consequently, imaging-based, histological (tissue-based) and serum-based biomarkers are the most frequently used biomarkers to assesses multiple aspects of BC management [4]. Nevertheless, tissue biopsy remains the primary source for tissue-based biomarkers, while liquid biopsy serves as a complementary, less invasive tool for accessing various circulating biomarkers in blood and other body fluids, including tumor cells, blood cells, exosomes, genetic material, or tumor-related proteins [5]. Imaging-based biomarkers, such as multiparametric breast magnetic resonance imaging (MRI), are considered indispensable in breast imaging practice, offering important information about tumoral tissue characteristics, and can be easily coupled with artificial intelligence (AI) applications [6]. Differential expression/concentration of molecules such as nucleic acids, proteins, and metabolites as well as intact cells are considered an accurate source of BC-associated biomarkers [3], which are classified as susceptibility/risk, diagnostic, prognostic, monitoring, prediction, pharmaco-dynamics/response, and safety biomarkers [7,8]. Even if there is a wide variety of biomolecules that are used as biomarkers, protein biomarkers are the most common type used in oncological theranostics and management of patients with BC [9].

The native sequence of an individual protein is determined by the genetic code, but genetic and epigenetic variations affect protein functions, changing the protein abundance, activity, specificity or affinity [10]. Proteins can adopt multiple spatial conformations, also changing their subcellular localization [11], stability, and protein–protein interactions (PPIs), so the proteome composition is highly dynamic and permanently reshaped under the action of various stimuli, specifically regulating and adapting all protein functions to their changing microenvironments [12,13,14,15]. Posttranslational modifications (PTMs) occur on proteins after translation and regulate their activities by modification of physico-chemical proprieties, localization, stability, activation, turnover, and PPIs [14,16,17]. PTMs are defined as reversible or irreversible alterations of existing proteins, usually generated by covalently adding small chemical groups to one or more amino acid residues in translated proteins [15]. Even if protein PTMs are reversible and commonly catalyzed by enzymes, some PTMs occur spontaneously and are known as reversible or irreversible non-enzymatic PTMs, including stress-induced modifications [18].

A total of 50–90% of human proteins undergo PTMs [19]. Thus, many PTMs contribute to proteome diversity and have been correlated with oncogenesis and cancer development [20], including BC, by dysregulated PTM pathways that contribute to abnormal cell proliferation during breast carcinogenesis [17,21]. Among conventional methods for PTMs analysis, immunohistochemistry, Western blotting, and protein microarray are still popular and efficient [22]. In conventional immunohistochemistry, the detection of PTMs was limited by the number of available antibodies [23], but recent advances in mass spectrometry (MS)-based proteomics/peptidomics have enabled researchers to investigate PTMs on a proteome-wide scale [14], complementing antibody-based techniques for the detection and quantitation of PTMs [24]. Hattori and Koide (2018) reported that the next-generation of high-quality antibodies to PTMs is challenging, but recent advances in protein engineering may generate highly functional PTM antibodies [25]. Modifications and interrelationships of multiple biomarkers must be considered [8]. Thus, Tang et al. (2022) emphasized that the newly emerged concept of dynamic network biomarkers based on fluctuations and correlation between various molecular groups is crucial for characterization of cancer initiation and progression [7].

More than 650 types of PTMs, such as phosphorylation, acetylation, methylation, ubiquitination, glycosylation, acylation, SUMOylation, neddylation, citrullination, succinylation, hydroxybuthyrylation, lactylation, carbamination, palmitoylation [26,27], deamidation, glycation, nitrosylation, and others, have been described [21,28,29]. Moreover, many regulators of PTMs, such as kinases, acetyltransferases, methyltransferases, ubiquitin ligases, oligosaccharyltransferases, and peptidyl arginine deiminases, can be considered as potential targets in various diseases, including BC [13]. Olzscha (2019) showed that the PTM-related enzymes can be classified as “writers” that add the PTMs to the substrate protein, “erasers” that can remove them, and “readers” that recognize the PTM, initiate a downstream signaling cascade, and exert a physiological response [30]. Thus, PTMs play important roles in regulating physiological processes, such as signal transduction, metabolism reprogramming, protein localization and turnover, as well as pathological processes [18,29], including many human hallmarks of cancer, with implications in cancer therapy [29]. PTMs can promote invasiveness and angiogenesis, metabolic reprogramming and suppression of anti-tumor immunity [31]. Additionally, PTMs have an important role in the shaping and regulation of the TME, which contributes to cancer progression [32].

PTMs of protein biomarkers are differentiated in different types of BC cells. For example, Zhou and Yu (2024) showed that histone PTMs, including acetylation, methylation, and phosphorylation, are differentially expressed in the luminal A BC subtype, compared to HER2+ and basal-like BC, being important for epigenetic regulation in TNBC [33]. Also, only in TNBC cell lines sensitive to signal transducer and activator of transcription 3 (STAT3) inhibition, such as BT-549 (mesenchymal-like), MDA-MB-231 (mesenchymal stem-like), and MDA-MB-468 (basal-like 1), was phosphorylated STAT3 at Tyr705 correlated with invasion and migration, while p-STAT3 inhibition has been shown to cause downregulation of matrix metalloproteinase 9 (MMP-9) and vimentin (VIM), both involved in invasion and migration [34]. On the contrary, Zhang et al. (2017) showed that, in both MDA-MB-231 (invasive TNBC, lacks estrogen receptor (ER) and progesterone receptor (PR) expression, as well as human epidermal growth factor receptor 2 (HER2) amplification) and MCF7 (luminal A molecular BC subtype, ER and PR positive) BC cell lines, deglycosylation of the epithelial cell adhesion molecule (EpCAM) promotes similar effects [35].

Reversible PTMs are often very dynamic, so the equilibrium between the modified and non-modified status of protein biomarkers can be changed, emphasizing adaptive bio-pathological effects on cancer cell fate [36]. Thus, both conjugation and deconjugation enzymes catalyze these dynamic and reversible PTMs, such as the process of SUMOylation [37]. The equilibrium between activation and inactivation of different signaling pathways is also controlled by phosphorylation/dephosphorylation mechanisms, involving protein kinase and phosphatase enzymatic activity [38] that modulates many cellular processes such as malignant transformation of cells, signal transduction, metabolism, protein localization, turnover and PPIs, stemness, survival, growth, proliferation, differentiation, invasion, EMT, and metastatic spread [39]. Moreover, the equilibrium between ubiquitination and deubiquitination reflects the balance between estrogen receptor alpha (ERα) degradation, through the ubiquitin proteasome system followed by ERα signaling deactivation, and ERα stabilization that promotes BC progression [40,41]. Additionally, histone acetylation modifications are also reversible, so BC may be treated by restoring histone-acetylation-based modifications to normal levels [42]. Consequently, histone acetyltransferases inhibitors and activators as well as histone deacetylase activators may be used as drugs to treat BC [42].

Tumor-associated antigens (TAAs) have elevated levels in tumor cells but are also expressed at lower levels in healthy cells, while tumor-specific antigens (TSAs) are proteins specifically expressed by tumors but not by normal cells. TAAs can suffer PTMs, often absent in normal proteins, resulting in abnormal protein structure and stability, folding, function/activity, and interaction with different molecular partners, including protein–protein interactions (PPIs) [20]. Consequently, aberrant PTMs can cause different types of proteinopathies [30]. Immunohistochemical biomarkers, including steroid hormone receptors, markers of proliferation and factors involved in epithelial–mesenchymal transition, apoptosis and angiogenesis, are used as prognostic and predictive factors or to direct treatment decisions in BC management [43]. Thus, ER, PR, androgen receptor (AR), HER2, Ki-67, and the tumor-suppressor protein p53 are the most common immunohistochemical BC prognostic and therapeutic biomarkers [43]. Immunohistochemical analysis of in situ AR phosphorylation [44], histone lysine acetylation [45], SUMOylation pathway [46] and breast cancer protein 1 (BRCA1) ubiquitination [47] showed that these PTMs are differentially expressed in BC tumorigenesis and progression, demonstrating their potential to reveal different grades, morphological types, phenotype classes of invasive BCs, cell-type-specific activation status and response to treatment with clinical significance, assessing, for example, the efficacy of kinase-targeted chemotherapies [48] or therapeutic modalities targeting protein acetylation [49].

In this review, we focused our analysis on the different types of PTMs of histological biomarkers in BC. Thus, we analyzed the most common PTMs, including phosphorylation, acetylation, methylation, ubiquitination, SUMOylation, neddylation, palmitoylation, myristoylation, and glycosylation/sialylation/fucosylation, of transcription factors (ERα, PR, AR, p53, and transcription factors involved in EMT), proliferation marker Ki-67, plasma membrane proteins, such as HER2 and cell adhesion proteins, and histone modifications. We emphasized that PTMs interfere with biomarker nuclear or subcellular localization, structure and function, stabilization or inactivation, initiation or silencing of genomic and non-genomic pathways including transcriptional activities or signaling pathways, proteostasis, cell–cell and cell–extracellular matrix (ECM) interactions, and PPIs. We concluded that the most common PTMs of usual biomarkers orchestrate all of the hallmark pathways that are dysregulated in BC. It is known that the number of clinical proteomics studies based on PTMs analysis is increasing [36]. Understanding protein PTMs in clinical samples and disease models allows for the development of biomarkers for the detection of BC and promotes the discovery of prognostic biomarkers and detection of novel potential therapeutic drugs targeting PTMs [21,36].

## 2. PTMs of Transcription Factors in Breast Cancer

Hormone receptors (HRs), including estrogen receptors (ERs), progesterone receptors (PRs), human epidermal growth factor receptor 2 (HER2), and androgen receptor (AR), are basic histological and molecular biomarkers used for the diagnosis and evaluation of therapeutic responses in female and male BC [50]. The proliferation index Kiel 67 (Ki-67, encoded by *MKI67* gene) protein is also an important biomarker for assessing tumor proliferating activity [4]. Histological biomarkers are generally detected using immunohistochemical techniques conducted on biopsy tissue samples [4]. ERs, PRs, and ARs are nuclear receptors and transcription factors (TFs). TFs play key roles in target gene expression, but are also associated with BC disease progression [51]. NRs are regulated by PTMs that impact their localization, receptor function, and PPIs. Many TFs are associated with epithelial–mesenchymal transition (EMT) and have been characterized as targets of SUMOylation and neddylation, known as crucial protein PTMs involved in tumorigenesis [52]. The following subchapters summarize how ERα phosphorylation can activate ERα signaling, how ERα and PR methylation regulates Erα–PR crosstalk, how ubiquitination leads to the degradation of ERα in the proteasomal system, and how deubiquitination stabilizes ERα and promotes BC progression. S-palmitoylation induces ERα localization to the plasma membrane, sustaining several signaling pathways involved in cell proliferation. PR phosphorylation activates PR-hormone-dependent transcription and targets p-PR for ubiquitination and proteasomal destruction in the cytosol. Methylation stabilizes PR, while SUMOylation attenuates PR activity on all target genes.

### 2.1. ERα

The estrogen receptor (ER) is a major classifier of BC as well as the first predictive biomarker in cancer [53]. There are three types of estrogen receptors that become activated when cells are exposed to estrogen: nuclear ERs, extra-nuclear ERs, and G-protein-coupled ERs (GPERs) [54]. Estrogen receptor alpha (ERα) and estrogen receptor beta (ERβ) are well known as two main nuclear receptors that mediate estrogen’s effects [55] and present several isoforms with incomplete functional domains resulting from alternative RNA splicing of encoding *ESR1* and *ESR2* genes, respectively [56]. Nuclear receptors are transcription factors that regulate the expression of targeted genes [57], and their activity is widely modulated by phosphorylation and other PTMs [58] that affect stability, sensitivity, activity and subcellular localization and are involved in BC carcinogenesis and progression [59]. Many authors have reviewed the most important PTMs of ERs, including phosphorylation, acetylation, methylation, ubiquitination, SUMOylation, and palmitoylation [54,59].

The main protein expressed by luminal cells is ERα [60], associated with both hormone-dependent and -independent cancer, which contributes to malignant progression or inhibition [54]. ERα is overexpressed in about 70% of BCs [61,62], so this receptor is critical for BC progression and is a central therapeutic target for hormone-dependent BC [63].

ERα protein expression of human BC is localized in the nucleus and/or membrane or cytoplasm, the ERα signaling pathways involving either classical nuclear/genomic effects or membrane/non-genomic actions that work together and in association with other hormones [63]. Thus, the canonical form of the ERα, the estrogen receptor alpha-66 (ERα66), is expressed in the nucleus, while its splice variant, the estrogen receptor alpha-36 (ERα36), is expressed at the cytoplasm/membrane level [60]. Moreover, the ERα46 is another truncated variant of the full-length ERα66, and it has been reported to have an inhibitory role in cancer cell growth [64]. Therefore, ERα66 and ERβ1 are estrogen nuclear receptors that mediate the estrogen-dependent pathological mechanisms in BC [55]. ERα controls the expression of proliferative genes in BC cells, so when bound by estrogens, ERα activity results in altered expression of target genes [61]. ERα66 and its related signaling pathway seem to be responsible for endocrine resistance induced by various mechanisms, including PTMs of ERα66 [65]. Thiebaut et al. (2020) also showed that different isoforms of nuclear ERα and ERβ can be located at the plasma membrane level following PTMs, such as palmitoylation or myristoylation [55]. For example, S-palmitoylation is a PTM known to affect the subcellular distribution and function of the modified proteins, promoting their translocation in the membranes [66]. Thus, the palmitoylation of ERα at Cys447 induces the localization of the receptor at the plasma membrane level [54]. Acconcia et al. (2005) demonstrated that the palmitoylation of ERα leads to its residence in the plasma membrane and interaction with caveolin-1 (CAV1) membrane protein in HeLa and HepG2 cell lines, followed by the initiation of several non-genomic activities, such as the activation of signaling pathways and E2-induced cell proliferation by AKT activation, cyclin D1 promoter activity, and DNA synthesis [67]. Rusidzé et al. (2021) showed that myristoylation is also a PTM that allows the residence of the ERα at the plasma membrane level [63]. Thus, ERα36 contains three potential myristoylation sites conserved in ERα66 and localized in the plasma membrane that could rapidly relay the estrogen signaling pathway, inhibiting the canonic transcriptional activity of ERα66, mainly due to the competition for DNA-binding sites [63]. In addition, the A/B domain located at the N-terminal end of ERα66 has several phosphorylation and SUMOylation sites that modulate the regulation of ERα66 target genes [55]. Approximately 30% of the human proteome is phosphorylated [15,68]. In protein phosphorylation, a phosphate group is chemically attached to specific amino acid residues of a protein, such as serine/threonine/tyrosine residues (Ser/Thr/Tyr) [69]. Thus, protein phosphorylation is the most common PTM involved in all cellular functions and pathological events, such as cell division and differentiation, protein degradation, cell signaling [70], gene transcription [11], autophagy [71], intercellular adhesion and communication, cell–ECM adhesion [72], epithelial–mesenchymal transition (EMT) [73], PPIs, and multidrug resistance [74]. In response to various stimuli, protein phosphorylation is modulated by the activity of kinases and phosphatases [75]. Consequently, in cancerous tissue, many signaling pathways and interaction networks are altered by protein phosphorylation [76], and MS-based proteomics/phosphoproteomics is the most important tool for phosphorylation analysis [75]. ERα regulates cell proliferation, differentiation and survival in BC, its activity being regulated by phosphorylation through various kinase signaling pathways [77], including c-Src kinase and the extracellular-signal-regulated kinase 1/2 (ERK1/2) signaling pathways [77], mitogen-activated protein kinase RAS-RAF (RAS-RAF-MAPK) cascade, p38 MAPK pathway, cyclin-dependent kinases CDK2 and CDK7, protein kinase C (PCK), pp90 RSK1, protein kinase B (AKT/PKB), IKKα kinase, and protein kinase A (PKA) [78]. The phosphorylation of the AF-1 domain induced by estradiol or/and growth factor signaling modulates the structure and function of ERα [79]. Castoria et al. (2012) reported that ERα phosphorylation at Tyr537 by Src regulates cytoplasmic localization of this receptor, and it was required for hormone responsiveness of DNA synthesis in the MCF7 BC cell line [80]. Further, Frei et al. (2016) demonstrated that the mediator of ERBB2-driven cell motility (MEMO), a redox-active protein with a key role in BC cell migration, invasion and metastasis, interacts with c-Src to control ERα extra-nuclear localization and Tyr537 phosphorylation [81]. Anbalagan and Rowan (2015) identified and characterized four novel ERα phosphorylation sites (S46/47, S282, S294, and S559) and showed that the proto-oncogene tyrosine-protein kinase Src (c-Src) signaling pathway impacts ERα phosphorylation and alters ERα function [77]. These authors also demonstrated that the disruption of ERα S118 and S167 phosphorylation in BC cells leads to significant effects on BC cell growth, morphology, migration, and invasion [77]. Interestingly, in BC models, overexpression of ERK1/2, Src activity, ERα phosphorylation, and tamoxifen resistance were correlated with light exposure at night [77]. In addition, ubiquitination of ERα was associated with its phosphorylation state, the interacting phosphorylation and ubiquitination of ERα controlling the quantity and function of this receptor [82].

Tecalco-Cruz and Ramirez-Jarquin (2018) showed that ERα polyubiquitination induces ERα degradation through the ubiquitin proteasome system [83]. However, these authors reviewed several studies that demonstrated that ERα polyubiquitination can be inhibited by PTMs among other mechanisms, followed by an increase in ERα protein levels and dysregulation of ERα signaling in BCs, sustaining malignant progression and resistance to endocrine therapy [83]. The ubiquitination of ERα and ERβ involves the carboxyl terminus of Hsc70-interacting protein (CHIP), the E3 ubiquitin-protein ligase MDM2 and ubiquitin ligase E6AP [82]. Conversely, Xia et al. (2019) showed that ubiquitin-specific protease 7 (USP7) interacts with ERα, mediating the deubiquitination and stabilization of ERα that promotes breast tumorigenesis [41]. In addition, the deubiquitinating enzyme USP15 also stabilizes ERα and promotes BC progression [40].

**Figure 1:**
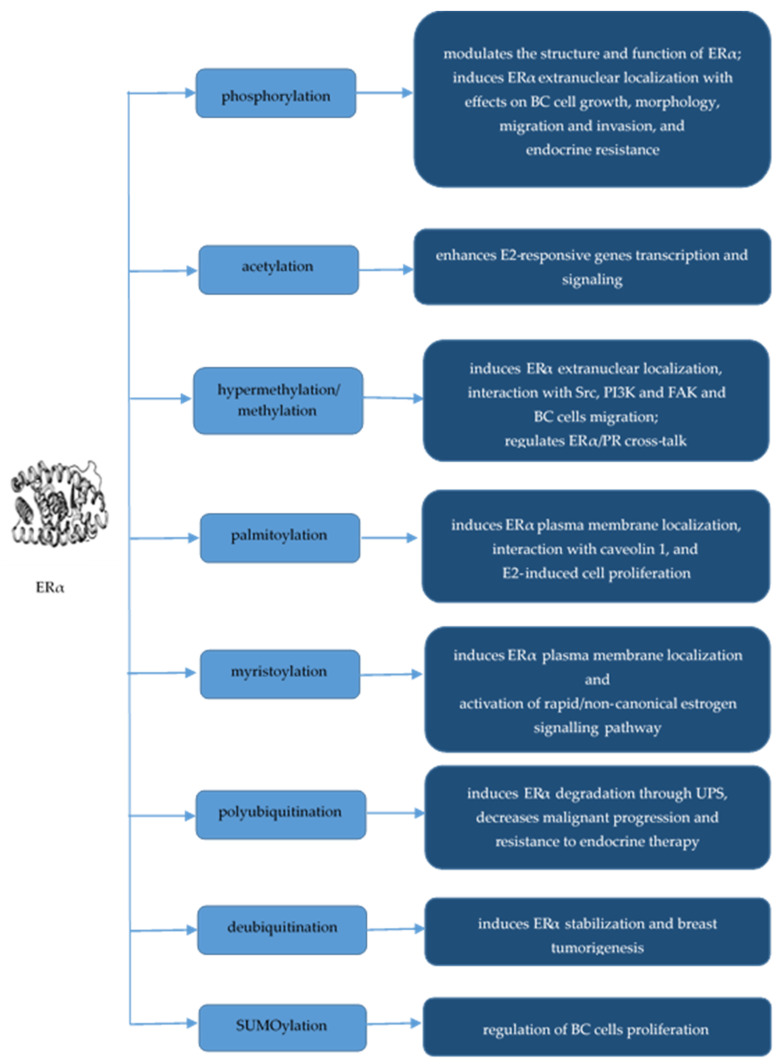
PTMs of ERα in BC.

SUMOylation is an essential protein PTM described as an important regulator of cell cycle, DNA repair and genomic stability, protein trafficking and turnover, cancer cell growth, proliferation, migration, and metastasis, which affects protein activity and subcellular localization [17,62]. SUMOylation involves the reversible attachment of a small ubiquitin-modifier (SUMO) protein isoform to an internal lysine residue of a target protein, modulating its stability, solubility, and PPIs [84]. Lin et al. (2021) concluded that elevated levels of SUMO1/2/3 can be associated with poorer overall survival and disease-free survival for TNBC patients, suggesting that MYC signaling in TNBC can induce the activation of SUMOylation pathway [46]. Additionally, Wang et al. (2023) showed that ERα-induced SUMO1 expression plays a significant role in the regulation of BC proliferation [62]. ERα, AR, glucocorticoid receptor (GR), PRs, mineralocorticoid receptor and peroxisome proliferator-activated receptor (PPAR) γ are regulated by SUMOylation [17]. Le Romancer et al. (2008) reported the hypermethylation of ERα in the cytoplasm of a subset of BCs through Arg260 methylation within the ERα DNA-binding domain by protein arginine methyltransferase 1 (PRMT1), an event that mediates the extranuclear function of ERα by triggering its interaction with PI3K and Src, involving the recruitment of a Src substrate, the focal adhesion kinase (FAK), which sustains BC cell migration [85]. The main PTMs of ERα in BC and their associated effects are listed in Figure 1.

### 2.2. PR

Classic nuclear isoforms of progesterone receptors (PR), PRA and PRB, mediate the genomic actions of progesterone [86], regulating differentiation and proliferation in normal mammary glands, but are also implicated in the pathogenesis of human BC [87]. Like ER, PRs are transcription factors from the nuclear receptor family. PRs modulate ERα action in BC cells and undergo PTMs, which include phosphorylation, acetylation, methylation, ubiquitination, and SUMOylation [17,88]. MAPK, CKD2, PKA or protein kinase CK2 phosphorylate PR at specific sites [88]. PRs are also SUMOylated on their N-terminal domain, so SUMOylation regulates PR auto-inhibition and trans-repression, and PR silencing is associated with the loss of its SUMOylation [17]. Chung et al. (2014) showed that PR acetylation at Lys183 by p300 acetyltransferase enhances PR activity by accelerated binding of its direct target genes, this effect being mediated by enhancing Ser294 phosphorylation [89].

### 2.3. AR

Expression of the androgen receptor (AR), also a ligand-dependent nuclear transcription factor, may serve as a prognostic and predictive biomarker in BCs [44], being detectable in about 90% of primary BC and 75% of metastasis [90]. PTMs of this transcription factor occur both in the cytoplasm and nucleus of the cell [90] and include phosphorylation, acetylation, methylation, ubiquitination, and SUMOylation [91]. Ren et al. (2013) showed that AR and its phosphorylation at Ser213/650 are differentially expressed in BC tumorigenesis and cancer progression; thus, phosphorylation at Ser213/650 was detected as increased in primary ER-negative BC, ductal carcinomas, and metastases compared to benign tissue and could have predictive value in BC prognosis [44]. pERK1/2 and pCDK1 phosphorylate AR in BC, so phosphorylation of AR at Ser515 has been identified as a potential prognostic marker for TNBC [90,92].

Additionally, Zhao et al. (2014) showed that AR is posttranslationally modified by hydrogen sulfide (H_2_S) via S-sulfhydration/persulfidation/sulfuration and that hydrogen sulfide (H_2_S) represses AR transactivation by targeting at the second zinc finger module [93]. Endogenous H_2_S is considered an oncogenic gas involved in tumorigenesis [94], due to its ability to induce angiogenesis, promoting tumor growth, EMT, protein persulfidation, and chemotherapy resistance, accelerating the cell cycle and inhibiting apoptosis [95,96]. In BC, exogenous H_2_S induces cellular acidification and inhibits the proliferation, migration, and invasion of MDA-MB-231 TNBC cells and decreased malignancy in TNBC xenograft tumor mouse models by dysregulation of genes and signaling pathways involved in cell cycle regulation and DNA replication pathways [97]. Shackelford et al. (2021) showed that H_2_S-synthetizing enzymes are overexpressed in BC among other malignancies [96]. Persulfidation by H_2_S, also called sulfhydration or sulfuration, is a key oxidative, reversible PTM of proteins [98,99], in which thiol groups in cysteine residues are converted into persulfides, H_2_S conveying its signaling and mediated biological functions [100,101]. Persulfidation alters the function, localization or stability of target proteins in a dynamically manner, regulating cell functions in response to stressor stimuli [99]. Thus, H_2_S mediates apoptosis in cancer cells through multiple signaling pathways involved in tumorigenesis and cancer development, such as PI3K/AKT/mTOR and MAPK [102]. Stoltzfus et al. (2024) showed that a plethora of zinc finger proteins are also targets of persulfidation [98].

The main PTMs of hormone receptor biomarkers in BC and their associated effects are listed in Table 1.

### 2.4. p53

The transcription factor p53, well-known as the “guardian of the genome”, is one of the most important tumor suppressors that facilitates DNA repair and genomic stability, cell cycle arrest, apoptosis, autophagy, senescence, and cellular metabolism [123,124]. In the presence of cellular stress, including hypoxia, oncogene activation, DNA damage, and nutrient deprivation, p53 undergoes PTMs, such as phosphorylation, ubiquitination, acetylation, methylation, SUMOylation, neddylation, glycosylation, and poly-ribosylation, so that dysregulated p53 can contribute to tumorigenesis [123,124,125,126].

p53 is a nuclear protein that comprises 393 amino acids, and about 50 amino acid residues may be modified [127]. Consequently, p53 PTMs are crucial for its localization, turnover, and stabilization [126]. In unstressed cells and tissues, p53 is present at a low level or latent state, maintained through its targeted degradation [128,129]. Many authors showed that phosphorylation leads to its translocation and accumulation into the nucleus and stabilizes p53 by disrupting its interaction with its negative regulators such as the oncoprotein mouse double-minute 2 homolog (MDM2), also known as E3 ubiquitin-protein ligase MDM2 [123] or HDM2 in humans [126]. In BC, the tumor-suppressive activities of p53 protein can be reduced by MDM2 overexpression or by its *TP53* encoding gene mutations present in 30–35% of all BCs [123]. Thus, genotoxic stress initiates signaling pathways that stabilize p53, cause it translocation and accumulation in the nucleus, and activate it as a transcription factor able to activate or suppress its targeted genes [129]. N-terminal phosphorylation is important for p53 stabilization and for acetylation of C-terminal sites, both PTMs mediating the response to cellular stress [129]. Phosphorylation of p53 at Ser46 by homeodomain-interacting protein kinase 2 (HIPK2) can lead to the transactivation of pro-apoptotic genes, such as p53 upregulated modulator of apoptosis (PUMA), in response to genotoxic stress, like DNA damage [126]. MDM2 binds and ubiquitinates p53, translocating it from the nucleus to the cytoplasm, leading to its degradation via UPS [124]. Previous dephosphorylation of p53 is necessary for its ubiquitination [126]. Neddylation is a PTM comparable with ubiquitination that conjugates a ubiquitin-like protein called neuronal-precursor-cell-expressed developmentally downregulated protein 8 (NEDD8) to a lysine residue in the target protein, modulating tumorigenesis by acting on tumor cells as well as tumor microenvironment (TME) components, such as immune cells, cancer-associated fibroblasts, and cancer-associated endothelial cells [130,131]. MDM2 and FOXO11 proteins facilitate the neddylation of p53, inhibiting its transcriptional activity [132]. Chauhan et al. (2020) emphasized that SUMOylation, which is also promoted by stress, regulates the stability and activity of the p53, mainly by SUMO1 and SUMO2, which modify p53 at lysine (K) 386, increasing its transcriptional activity, promoting apoptosis and inducing p53-dependent cell senescence [133]. These authors showed that SUMO-specific peptidase 1 (SENP1), a SUMO-specific protease with pro-tumorigenic roles, deSUMOylates p53 in vivo and in vitro, SENP1 silencing inducing p53 transactivation activity [133].

Acetylation is the process of covalently binding acyl-CoA compounds to specific amino acid sites of proteins, usually to lysine (abbreviated as Lys or K) residues [49]. Protein acetylation is a reversible PTM involved in many cellular processes, such as DNA damage repair, chromatin remodeling, transcriptional regulation, and energy metabolism, often associated with cancer [49]. Thus, protein acetylation plays both oncogenic roles in BC, promoting EMT, invasion and metastasis, cell proliferation and survival as well as BCSC characteristics and inhibiting sensitivity of cancer cells to anti-tumor chemotherapy as well as tumor-suppressive roles through maintenance of ubiquitin-mediated degradation of oncogenic acetylated proteins, inhibition of BCSC reprogramming and self-renewal, inhibition of EMT, migration and invasion of cancer cells, and increasing the cytotoxicity of chemotherapeutic drugs [21]. Nagasaka et al. (2022) showed that p53 acetylation plays a key role in p53 regulation, being an indicator of its activation [124]. In addition, many lysine residues may be acetylated, promoting the DNA binding of p53 and increasing its transcriptional activity, but the acetylation of these sites may concomitantly impair the ubiquitination by MDM2, blocking the degradation of p53 and inducing its accumulation [124]. p53 acetylation/mutation at specific sites, essential for the regulation of p53 metabolic targets, may lead to failure of p53-mediated ferroptosis, induction of apoptosis by upregulation of the expression of pro-apoptotic genes such as PUMA, and induction of p53-mediated cell cycle arrest [124]. Liu et al. (2021) demonstrated that p53 function is regulated by protein arginine methyltransferase (PRMT1) in BC cells [134]. Thus, PRMT1 silencing activates the p53 pathway and induces cell growth arrest and senescence, while PRMT1 binding to p53 inhibits its transcriptional activity [134].

**Figure 2:**
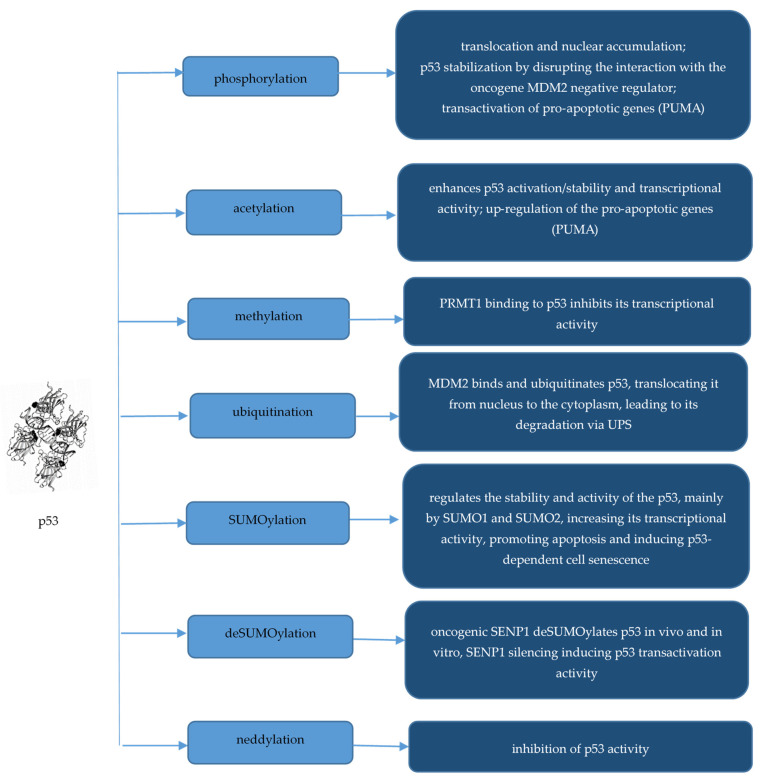
PTMs of p53 tumor-suppressor protein in BC.

The most important PTMs of p53 tumor-suppressor protein in BC are summarized in Figure 2.

### 2.5. Transcription Factors Involved in Epithelial–Mesenchymal Transition

Transcription factors (TFs) are primary regulators of gene expression, binding specific DNA sequences and thus regulating gene expression [135]. TFs activate the transcription of cytokine genes and undergo PTMs to dynamically adapt cytokine production for maintenance of immune balance [136]. EMT, a critical step in metastasis and cancer development, requires the regulation of specific proteins by EMT-inducing transcription factors, such as TWIST1, SNAIL, SLUG, and ZEB proteins, so that phosphorylation, ubiquitination, acetylation, methylation, glycosylation, SUMOylation and other PTMs of these proteins induce activation/destabilization of EMT-inducing transcription factors and nuclear/cytoplasmic accumulation, promoting EMT or, on the contrary, mediating their ubiquitination and proteasomal degradation after their translocation in the cytoplasm. EMT is also associated with characteristics of breast cancer stem cells (BCSCs), including chemotherapy-resistance [137]. Thus, PTMs regulate multiple function of TFs, including their stability, localization, PPIs, and degradation [138].

TWIST1 is a major TF that regulates transcription during embryonic development and promotes EMT on BC cells, invasion, and metastasis, playing an essential role in tumor initiation, angiogenesis, stemness, and resistance to chemotherapy [139]. TWIST1 can be phosphorylated at Ser68 by p38, c-Jun N-terminal Kinases (JNK), ERK1/2, and MAPKs, leading to TWIST1 stabilization, EMT, invasiveness, and metastasis in BC [140]. TWIST1 phosphorylation by AKT1 is required for beta-transducin repeat-containing protein (β-TrCP)-mediated TWIST1 ubiquitination and proteasomal degradation, while protein kinase B (PKB alpha or AKT1) silencing stabilizes TWIST1 and enhances EMT in BC cells [141].

Zinc finger E-box binding homeobox 1 (ZEB1) transcription factor is frequently expressed in carcinomas, being involved in normal development and disease by TGFβ-induced responses and EMT [142]. ZEB1 PTMs, such as phosphorylation, acetylation, ubiquitination, and SUMOylation, influence ZEB1 half-life, its subcellular localization, and DNA/protein binding ability [16]. Llorens et al. (2016) showed that a decrease in ZEB1 phosphorylation increases both DNA/protein binding and transcriptional repression of ZEB1 target genes [142]. Consequently, PTMs of ZEB1, a repressor of epithelial genes, such as *CDH1* gene encoding E-cadherin, could contribute to the regulation of ZEB1-targeted gene expression [16]. DNA-sensing kinase ataxia-telangiectasia mutated ATM phosphorylates and stabilizes ZEB1 in response to DNA damage; ZEB1 interacts with ubiquitin-specific peptidase 7 (USP7) and enhances its ability to deubiquitinate and stabilize checkpoint kinase 1 (CHK1), promoting DNA repair and resistance to radiation [137].

Zinc finger protein SNAI1 (also referred to as SNAIL) is a TF upregulated in cancer, which acts as a regulator of EMT in BC cells by repressing E-cadherin expression [143,144]. SNAIL phosphorylation can emphasize opposite effects: phosphorylation on Ser246 by p21-activated kinase 1 (PAK1) leads to its nuclear accumulation and promotes EMT, while phosphorylation on Ser11 by protein kinase D1 (PDK1) induces the nuclear export of SNAIL, as well as its ubiquitination, degradation, and EMT inhibition [143,145]. GSK3β also phosphorylates SNAIL to regulate its β-TrCP-mediated ubiquitination and proteasomal degradation at a subcellular level [144,146]. In addition, Wnt/β-catenin signaling inhibits SNAIL phosphorylation, increasing SNAIL protein levels and activity and, consequently, driving in vivo EMT [146]. SNAIL silencing in MDA-MB-231 and T47D BC cells reduces or relocalizes p-ERK from the nucleus to the cytoplasm, while SNAIL overexpression in MCF7 BC cells induces EMT, migration, tumorigenicity and decreases cell adhesion [143]. SNAIL phosphorylation by activated ERK2 at Ser82/104 [147] and large tumor suppressor kinase 2 (LATS2) at Thr203 [148] leads to SNAIL nuclear localization, increasing its stability and activity, and allows it to escape from TRCP-mediated polyubiquitination and degradation, facilitating BC metastasis [138].

Signal transducer and activator of transcription 3 (STAT3) is a useful biomarker for early BC and promotes BC malignancy via resistance to apoptosis [149]. Phosphorylated STAT3 (pSTAT3) sustains cancer cell proliferation by overexpression of anti-apoptotic proteins (Bcl-2, Bcl-xl), pluripotency markers (transcription factor OCT4), and proto-oncogenes (MYC) [150]. Proietti et al. (2004) showed that progestins induce transcriptional activation of STAT3 via phosphorylation of STAT3, Jak1/Jak2 and c-Src [151].

GATA3 is a transcription factor often overexpressed in BC that regulates BC progression with context specificity, so its PTMs, such as phosphorylation, acetylation, methylation and ubiquitination, are critical to its function [152]. In lung adenocarcinoma, GATA3 is acetylated at K119 by acetyltransferase CBP and is deacetylated by HDAC1, HDAC2, and HDAC3; GATA3 acetylation inhibits cell migration and invasion, downregulating EMT-related transcription factors SLUG, ZEB1, and ZEB2 [152].

Dai et al. (2014) showed that PTMs of SRY-box transcription factor 2 (SOX2), OCT4, Krűppel-like factor 4 (KLF4), and c-MYC are involved in the generation of induced pluripotent stem cells (iPSCs) by reprogramming adult cells, regulated by protein kinase B (PKB/AKT) [153]. It is well known that stem/progenitor cells, also named tumor-initiating cells (TICs), are essential for BC carcinogenesis and progression [154]. AKT directly phosphorylates OCT4 to modulate OCT4/SOX2 dimer formation and facilitates the P300-mediated acetylation of OCT4, SOX2, and KLF4, or indirectly stabilizes KLF4 by inactivating GSK3 [153]. These authors suggest that AKT inhibition could target cancer stem cells [153]. The most cited PTMs of transcription factors involved in BC EMT are characterized in Table 2.

## 3. PTMs of Proliferation Marker Ki-67

Ki-67, a nuclear non-histone protein with cell-cycle-specific behavior [162], is one of the most commonly used protein biomarker for identifying all proliferating somatic cells in tumors [163] due to its important role in cell division [164]. Remnant et al. (2021) showed that hyperphosphorylation or mitosis-specific phosphorylation influences the changes in Ki-67 localization when cells transition from G2 to mitosis and Ki-67 remains phosphorylated in all active phases of cell cycle [163]. Endl and Gerdes (2000) demonstrated that Ki-67 phosphorylation by CDC2 kinase and protein kinase C (PKC) accompanies a specific translocation from the interior of the nucleus or nucleoli to the chromosome periphery during mitosis [162,165].

## 4. PTMs of Plasma Membrane Proteins

Plasma membrane proteins play key roles in cell signaling processes, including cell–cell and cell–ECM interactions that orchestrate the reception of various external stimuli and transmit them to the interior of the tumoral cell [166]. Ziegler et al. (2014) demonstrated that BC cells emphasize multiple strategies to sustain BC cell growth, reflected by aberrant expression of tyrosine kinases (i.e., HER2), cellular adhesion molecules, and structural proteins from the plasma membrane that could be considered as potential targets for BC theranostics [167].

### 4.1. HER2

*HER2* amplification occurs in 20–25% of BCs and results in overexpression of the ERBB2 receptor (HER2), a transmembrane glycoprotein with tyrosine kinase activity [168]. Interestingly, in HER2-negative BC cell lines, activation through phosphorylation of HER2 made this biomarker recognizable by trastuzumab, a chemotherapeutic drug that targets the extracellular domain of the HER2 protein [168,169]. Xia et al. (2023) reported that the neddylation of HER2 is a novel PTM that controls its expression and oncogenic activity in BC [170]. These authors demonstrated that neddylation inhibition promotes the degradation of HER2 protein by improving its ubiquitination, while HER2 neddylation promotes tumor growth in BC [170].

The localization, accumulation, secretion, stability, and function of hundreds of proteins in the cell can be altered by palmitoylation, a reversible protein PTM [171,172]. Protein S-palmitoylation is a type of PT lipidation, forming a reversible thioester bond between a cysteine residue and palmitic acid; N-palmitoylation occurs when a fatty acid palmitate is linked to cysteine at the N-terminal of a protein by a stable amide bound, while O-palmitoylation is the covalent attachment of palmitic acid to serine and threonine [172,173]. Addition of a lipid chain to proteins increases their hydrophobicity as well as stability, interactions, localization and membrane trafficking [174]. As in other cancer types, in BC, numerous proteins encoded by both oncogenes (i.e., EGFR, PD-L1) or tumor-suppressor genes, such as scribble (SCRIB), are palmitoylated, leading to modification in their cell localization, PPI, and signal transduction, sustaining cell proliferation, metastasis, and tumor immunity [175]. For example, EGFR is a glycoprotein expressed as a transmembrane tyrosine kinase receptor that can be internalized in the nucleus of the cell, acting in gene transcription, increasing proliferation, and favoring resistance to chemotherapy [171]. A study conducted by Bollu et al. (2014) showed that EGFR also exists in the mitochondria of BC cells as mtEGFR, where, activated by EGF, it induces palmitate synthesis that, in turn, activates mtEGFR through its palmitoylation that promotes mitochondrial fusion and cell survival [27]. The most significant PTMs of HER2 and EGFR biomarkers in BC are summarized in Table 3.

### 4.2. Cell Adhesion Proteins

Efficient cell adhesion and migration is necessary for efficient dissemination of tumor cells [180]. To regulate cancer cell–cell and cell–ECM adhesion, the epithelial cell adhesion molecule (EpCAM) forms a complex to integrin β-1 (ITGB1) [181]. EpCAM, a multifunctional transmembrane glycoprotein localized in the basolateral plasmalema of normal epithelial tissues, is frequently overexpressed in BC [182]. EpCAM is known as an adhesion molecule and a biomarker for circulating tumor cells, involved in the mediation of intercellular adhesion, proliferation, migration, stem cell characteristics, and EMT of cancer cells [183]. Integrin mediates cell–cell and cell–ECM interactions and promotes invasion and migration of BC cells, connecting ECM molecules to the intracellular actin skeleton [184]. Glycosylation is the most abundant and diverse PTM of proteins [185], an important regulator that plays key roles in modulating protein activity [186], including protein folding, stability, PPIs, and stress-related signaling processes [187]. Aberrant glycosylation is a significant characteristic of cancer development [188], mainly due to the role of glycosylation/sialylation of cell surface proteins in the regulation of apoptosis [35]. Thus, EpCAM glycosylation has been associated with its function; N-glycosylation mutation of EpCAM downregulates adhesion capacity through the FAK/AKT/GSK3β/β-catenin signaling pathway and MMP2/9 expression [187]. Zhang et al. (2017) showed that, in MDA-MB-231 and MCF7 BC cell lines, deglycosylation of EpCAM promoted apoptosis, downregulating the expression of anti-apoptotic protein Bcl-2 and overexpressing the expression of pro-apoptotic proteins Bax and caspase-3 through the ERK1/2 and JUK/MAPK signaling pathways [35]. Liu et al. (2017) showed that EpCAM promotes the activation of integrin β-1 (ITGB1), enhancing BC cell adhesion to fibronectin [187]. Yeh et al. (2017) emphasized the regulation of cavelolin 1 (CAV1) and ITGB1 modulated by matrix stiffness, which reciprocally control each other, CAV1 being involved in integrin-dependent signaling [189]. CAV1 is a multifunctional membrane protein involved in endocytosis, plasma membrane assembly, signal transduction, transport across the plasma membrane, cholesterol homeostasis, lipid transport and storage, cell cycle, proliferation, apoptosis, autophagy, cancer cell invasion, migration, and metastasis [190]. Han et al. (2023) showed that CAV1 acts as a tumor suppressor in BC cells [184], its expression being more closely associated with basal-like BCs [191]. A mechanism to reverse multidrug resistance in drug-resistant BC cells is based on the receptor for activated C kinase 1 (RACK1) activity that mediates the binding of Src, a P-gp-binding protein, to the overexpressed P-glycoprotein (P-gp) and modulates the activity of this drug pump, able to efflux anticancer drugs through regulation of the phosphorylation of caveolin-1 (CAV1), a protein that modulates P-gp activity, by Src [74].

Tumor cells express high levels of sialic acid [192]. Sialylation is a type of glycosylation that occurs on cell surface proteins, lipids and glycoRNAs, accumulating in tumors and helping cancer cells to survive and avoid immune surveillance and thus invade and migrate [193]. Playing a crucial role in cell–cell adhesion, hypersialylation, an increased expression of sialic-acid-terminated glycoconjugates on the cell surface [188], and overexpression of sialyltransferases are known as hallmarks for several cancers, including BC [194]. Alteration in ITGB1 sialylation is a regulator of cell adhesion and invasion of BC cells to ECM [195], so integrins have been reported as highly α2,6 sialylated on the MDA-MB-231 BC cell line but not on MCF7 BC cells [195]. Integrin β4 (ITGβ4), a transmembrane receptor for basal membrane and ECM components, plays a significant role in BC invasion, proliferation, EMT, and angiogenesis [196]. Tai et al. (2015) emphasized that the epidermal growth factor receptor (EGFR)/Src signaling triggers the tyrosine phosphorylation of ITGβ4, which recruits the focal adhesion kinase (FAK) to ITGβ4 and induces FAK activation and signaling [72]. Thus, EGFR/Src-dependent ITGβ4/FAK complex contributes to malignancy of BC [72].

E-cadherin is currently used to differentiate between lobular carcinomas, without immunohistochemical expression of E-cadherin, and ductal carcinomas, which express cytoplasmic E-cadherin, in cases with non-specific morphological characteristics [43]. Invasive lobular BC, but also high-grade ductal carcinomas, show reduced/loss levels of E-cadherin, a calcium-dependent cell–cell adhesion protein and a marker of phenotypic plasticity encoded by the tumor-suppressor gene *CDH1* [197]. Pinho et al. (2012) showed that, during EMT and its reverted process, mesenchymal–epithelial transition (MET), E-cadherin can be significantly targeted and regulated by glycosylation catalyzed by N-acetylglucoaminyltransferase-III (GnT-III) [198].

Scribble (SCRIB) is a cell junction and tumor-suppressor protein with an important role in maintaining polarity of epithelial cells [173]. SCRIB is overexpressed and mislocated in human cancers, being involved in the regulation of Hippo pathway in BC cells [173]. Chen et al. (2016) found that zinc finger DHHC-containing protein ZDHHC7-mediated SCRIB S-palmitoylation is important for SCRIB membrane trafficking, cell polarity, and tumor suppression, emphasizing the importance of palmitoylation for cell polarity and tumorigenesis [173]. Coleman et al. (2015) showed that curcumin inhibits the palmitoylation of ITGβ4 in invasive BC cells that overexpress ITG palmitoylation [26].

### 4.3. Other Proteins

Fucosylation is a type of glycosylation that can induce increased invasion and metastatic potential in BC [31]. For example, fucosylated clusterin (CLU), a glycoprotein known as apolipoprotein J (APOJ), implicated in apoptosis, cell cycle regulation and DNA repair [199], represents a luminal BC-associated glycoform that can play a role in BC progression via pro-angiogenic cytokines and TNF-α production by intratumoral macrophages [200]. It is known that the activity of CLU, a molecule that acts as a chaperone, depends on its degree of glycosylation [201].

Programmed cell death ligand 1 (PD-L1) was found to be overexpressed in BC as well as in other types of solid tumors, and it is involved in tumor progression, cell proliferation and survival [202]. PD-L1 is a transmembrane protein known for its oncogenic function exerted by activation of Wnt/β-catenin pathway [203,204]. Palmitoylation occurs on PD-L1, affecting its stability, so targeting PD-L1 palmitoylation sensitizes tumor cells to T-cell killing and inhibits tumor growth [203]. Most cellular proteins become ubiquitinated during their lifespan [205]. Thus, ubiquitination is one of the most important types of PTM, involved in many cellular activities through the regulation of protein degradation [206]. Tens of thousands of ubiquitination sites on thousands of proteins have been identified [205]. The expression level of programed cell death-1 (PD-1) and its ligand PD-L1 may be regulated by the ubiquitin–proteasome system (UPS), the primary site of ubiquitination [202]. PTMs of cell adhesion and other membrane proteins are listed in Table 4.

## 5. Role of Histone Modifications in BC

Zhuang et al. (2020) showed that dynamic and reversible histone modifications (HMs), mainly acetylation and methylation, are significant epigenetic alterations during BC progression [212]. Moreover, evidence suggests that phosphorylation and acetylation can emphasize synergistic coupling, as in the case of histone H3 in response to EGF stimulation, when H3 phosphorylation affects the efficiency of subsequent acetylation [213]. Elsheikh et al. (2009) used immunohistochemistry and Western blotting for detection, semi-quantification and validation of HMs in BC, including histone lysine acetylation (H3K9ac, H4K12ac, and H4K16ac), lysine methylation (H3K4me2 and H4K20me3) and arginine methylation (H4R3me2), correlated with tumor phenotypes, prognostic factors, and patient outcome [45]. This analysis revealed low or absent H4K16ac expression in the majority of analyzed BC cases, suggesting that this modification could represent an early sign of BC, moderate or low levels of lysine acetylation or lysine and arginine methylation being detected in basal carcinomas and HER2+ BCs [45].

Thus, HMs are involved in genomic stability, DNA repair, transcription, and the modulation of chromatin remodeling in BC cells [214]. HM enzymes involved in cancer metastasis are histone acetyltransferases (HATs) and histone deacetylases (HDACs) as well as DNA methyltransferases (DNMTs) [212]. Histone acetylation is catalyzed by HATs as writer enzymes, such as Gcn5-related N-acetyltransferases (GNATs), MYST and ORPHAN (p300/CBP) families, while HDACs act as eraser enzymes, both of them controlling the active or silent state of chromatin and changing the transcriptional activity of genes [212,214]. Thus, acetylated chromatin becomes loose and can be related to activated transcription, while deacetylated chromatin is supercoiled and could be associated with the inhibition of the expression of tumor-suppressor genes in BC [212] like breast cancer susceptibility gene 1 (BRCA1), a tumor-suppressor gene involved in DNA repair, cell cycle, and genome stability [215]. HDAC class III sirtuins are histone deacetylases found to be overexpressed in BC, regulating various proteins involved in chromatin remodeling, energy metabolism, inflammation, cell proliferation, apoptosis, autophagy, chemoresistance, invasion, migration, and metastasis [154,212,216]. Du et al. (2020) showed that mitochondrial sirtuin SIRT4 exerts its tumor suppressive function in BC, while its loss promotes self-renewal of breast cancer stem cells (BCSCs) through the modulation of SIRT1 expression, which regulates the acetylation of histones H4 and cell stemness via BRCA1 [154,215].

## 6. Conclusions

Proteins are the most common types of biomarkers used in BC theranostics and management. A disease biomarker must be a relevant, objective, stable, and quantifiable biomolecule or other parameter, but proteins are known to exhibit the most varied and profound structural and functional modifications. Thus, the proteome is highly dynamic and permanently reshaped and readapted, according to the cell or tissue changing microenvironments, trying to maintain local homeostasis. In this review, we focused our analysis on the different types of PTMs of histological biomarkers in BC. Thus, we analyzed the most common PTMs, including phosphorylation, acetylation, methylation, ubiquitination, SUMOylation, neddylation, palmitoylation, myristoylation, and glycosylation/sialylation/fucosylation, of transcription factors (ERα, PR, AR, p53, and transcription factors involved in epithelial–mesenchymal transition), proliferation marker Ki-67, plasma membrane proteins, such as HER2 and cell adhesion proteins, and histone modifications. Most of these PTMs occur in the presence of cellular stress, mainly genotoxic stress, including hypoxia, oncogene activation, DNA damage, and nutrition deprivation. PTMs correlate with cellular and molecular morphological modifications, including changes in cancer cell morphology by the formation of invadopodia, mitochondrial fusion, and chromatin remodeling, as in the case of acetylated chromatin, which becomes loose and can be related to activated transcription, while deacetylated chromatin is supercoiled and could be associated with the inhibition of the expression of tumor-suppressor genes in BC.

We emphasized that these PTMs interfere with biomarker maintenance, turnover and lifespan, nuclear or subcellular localization, structure and function, stabilization or inactivation, initiation or silencing of genomic and non-genomic pathways including transcriptional activities or signaling pathways, mitosis, proteostasis, cell–cell and cell–extracellular matrix (ECM) interactions, membrane trafficking, and PPIs. Moreover, PTMs of usual biomarkers orchestrate all hallmark pathways that are dysregulated in BC, playing both pro- and/or antitumoral context-specific roles in DNA damage, repair and genomic stability, inactivation/activation of tumor-suppressor genes and oncogenes, phenotypic plasticity, epigenetic regulation of gene expression and non-mutational reprogramming, proliferative signaling, endocytosis, cell death, dysregulated TME, invasion and metastasis, including epithelial–mesenchymal/mesenchymal–epithelial transition (EMT/MET), and resistance to therapy or reversal of multidrug therapy resistance.

PTMs of classical BC biomarkers can exert opposite effects on one biomarker, as in the case of TWIST1, when phosphorylation by MAPKs leads to its stabilization, invasiveness, EMT, and metastasis, while its phosphorylation by AKT1 is required for its ubiquitination and proteasomal degradation. SNAIL phosphorylation can also emphasize opposite effects: phosphorylation by PAK1 leads to its nuclear accumulation and promotes EMT, while phosphorylation by PDK1 induces the nuclear export of SNAIL, its ubiquitination, degradation, and EMT inhibition. Several types of PTMs may act synergistically, such as phosphorylation and acetylation of histones, PR and p53 tumor-suppressor protein. Many PTMs occur in the cytoplasm as well as in nucleus and induce biomarker translocation with opposite effects. According to Geffen et al.’s (2023) pan-cancer analysis, the most cited PTMs of these well-known biomarkers in BC are serine/threonine phosphorylation and lysine acetylation [217]. In conclusion, these well-known biomarkers used for BC diagnosis and classification harbor multiple PTMs and act together to reshape and adapt the BC cell proteome, according to a changing microenvironment. Analysis of in situ PTMs of histological biomarkers in BC correlating with tumor phenotypes, prognostic factors and patient outcome may have clinical significance, and allows for the discovery and validation of new biomarkers in BC, mainly for early diagnosis.

## Figures and Tables

**Table 1:** PTMs of hormone receptor biomarkers in BC.

Hormone Receptors	Role	PTMs	Modifying Enzymes	PTM Sites	Effects of PTMs
Estrogen receptor alpha (ERα encoded by *ESR1* gene)	nuclear receptor; transcription factor involved in cell proliferation, differentiation, and homeostasis; related to risk of BC, EC, and OC [77]; expressed in 70% of BC cases [41]	phosphorylation	c-Src kinase and ERK1/2 signaling pathways [77]	Ser118/167 [77]; Tyr537 [80,81]	alteration of ERα function (chromatin interaction, gene expression, BC growth, morphology, migration, invasion, and response to endocrine therapy) [77]; ERα phosphorylation by Src regulates its hormone-dependent nuclear export and cell cycle progression in BC cells [80]
			RAS-RAF-MAPK cascade [103]	Ser118 [103]	ER activation [103]
		acetylation	lysine acetyltransferases p300/CBP [104]	Lys266/268	enhances E2-responsive genes transcription and signaling in BC [104,105]
		methylation	PRMT1	Arg260 [85]	regulates ERα/PR cross-talk and activates a signaling complex in aggressive human BC [106]
		ubiquitination	ubiquitin ligases (CHIP, E3 ubiquitin-protein ligase MDM2, and E6AP)	K/Lys48	degradation of ERα in UPS, changes in ERα function [82]
		deubiquitintion	deubiquitinases USP7 [41], USP1 [107], USP15 [40], USP14 [108], MINDY1 [109]	K/Lys48-linked ubiquitination	stabilizes ERα/prevents ERα degradation in BC and promotes BC progression in ERα+ BC [40,41] and EC [108], inhibits ERα K48-linked poly-ubiquitination [107]
		SUMOylation	SUMO1 [110]	SUMOylated lysines are located in the hinge region of ERα [110]	regulates ERα-dependent transcription in MCF7 BC cell line [110]; full anti-estrogenicity in the absence of accelerated receptor turnover [111]
		S-palmitoylation	palmitoylacyltransferases ZDHHC-7 ZHHHC-21 for sex steroid hormones	Cys447	induces ERα localization to the plasma membrane and ERα-CAV1 interaction, enabling rapid E2 signaling for cell proliferation by ERK/MAPK, PI3K/AKT activation [67]
Progesterone receptor (PR)	nuclear receptor; transcription factor; modulates ERα action in BC; activates cytosolic signaling pathways; upregulated target gene of ER; prognostic biomarker in BC, especially in HR+ BC [88,112]; PR-A is involved in cell adhesion, morphology, invasiveness and metastasis, resistance to apoptosis and tamoxifen; PR-B is required for mammary gland development and expansion [113]	phosphorylation	MAPK CDK2 PKA CK2 EGFR-induced phosphorylation/ERK-mediated, c-Src [112,114]	Ser294/345 [112,115]; Ser190/294/554/676 [116]; Ser191A [117]	regulates PR cell cycle and growth-promoting target genes [112]; activates PR hormone-dependent transcription [116]; PR phosphorylation targets it for ubiquitination and proteasomal destruction in cytosol [114]
		acetylation	p300 acetyltransferase	Lys183	enhances PR-dependent initiation and reinitiation process via accelerated binding of its direct target genes; enhances Ser294 phosphorylation levels and PR activity, suppressing SUMOylation of PR [88]
		methylation	PRMT1	Arg637	stabilizes PR and accelerates its recycling and transcriptional activity; methylate ERα^Arg260^, involved in ERα/PR cross talk [106]
		ubiquitination	PR phosphorylation via EGFR pathway leads to PR ubiquitination [114]		EC cells undergo loss of PR and do not respond to progestin therapy [114]
		SUMOylation/ deSUMOylation	SENP1 deSUMOylates PRA		SUMOylation attenuates PR activity on all target genes [113]
		palmitoylation	palmitoylacyltransferases ZDHHC-7 ZHHHC-21 for sex steroid hormones	a small fraction of the PR is palmitoylated and anchored to the cell membrane (mbPR), into a complex with ERα [118]	mbPR, upon progestin exposure, activates the Src/RAS/ERK kinase pathway, leading to phosphorylation of nPR by the ERK signaling pathway, leading to intracellular PR phosphorylation, gene regulation, and entry into cell cycle in the absence of detectable intracellular progestin [118]
Androgen receptor (AR)	ligand-dependent/ligand-independent nuclear transcription factor, detected in 90% of primary BC, and 75% of metastasis [90]; prognostic and predictive biomarker in BC [44], involved in transcription, cellular proliferation, apoptosis in prostate cells [119]	phosphorylation	ERK1/2 and CDK1 [90]	increased on Ser213/650 in primary ER-negative BC, ductal carcinomas and metastases, compared to benign tissue [44]; Ser515 [92]	role in signal transduction; predictive value in BC prognosis [44]
			PI3K/AKT signaling [120]	Ser 213/791	AKT is an activator of AR required for HER2 signaling to androgen-independent survival and growth of PC cells [120]
				Ser210/790	AKT suppresses androgen-induced apoptosis by phosphorylating and inhibiting AR [121]
				Ser16/81/256/308/424/650/94 in COS1 fibroblast-like cells and LNCaP cells [122]	analysis of cross-talk between growth factor signaling and androgen in prostate development, physiology, and cancer [122]
		acetylation	KATs (CBP, P300, PCAF, TIP60, MOF, HBO1 ARD1) [119]		plays an important role in directing its cellular activities, modulating its stability, nuclear localization, and transcriptional activity [119]

**Table 2:** PTMs of transcription factors involved in EMT.

Transcription Factors	Role	PTMs	Modifying Enzymes	PTM Sites	Effects of PTMs
TWIST1	regulator in embryonic development; implicated in tumor initiation, stemness, angiogenesis, spreading, and chemoresistance; promotes EMT in BC [139]	phosphorylation	MAPKs, JNK, ERK1/2, p38	Ser68	induces TWIST stabilization, EMT, invasiveness, metastasis in BC [140]
			AKT1	Ser42/123, Thr121	sustains β-TRCP-mediated TWIST1 ubiquitination and degradation; suppresses EMT; AKT1 inhibition stabilizes TWIST1 and enhances EMT in BC cells [141]
SMAD3		phosphorylation	MAPKs, CDKs, GSK3β		enhances or suppresses TGF-β signaling involved in apoptosis, growth arrest, differentiation, and EMT [73]
Zinc finger E-box binding homeobox 1/2 (ZEB1/2)	frequently expressed in carcinomas, being involved in normal development and tumor metastasis [142]	phosphorylation	IGF1/MEK/ERK pathways; PKC	Thr867 hr851, Ser852, Ser853	ZEB downregulation/transcriptional repression of ZEB1 [16]
		autophosphorylation	ATM hyperactivation	Ser585	ZEB1 upregulation in radioresistant BC cells
SNAIL/SNAI1 transcriptional repressor 1	plays a role in the control of EMT and fibroblast activation [155]	phosphorylation	PAK1	Ser246	induces SNAIL activation and accumulation in the nucleus; promotes EMT [143]
			PI3K	Ser246	increased SNAIL activation [156]
			PKD1	Ser11	nuclear export (destabilization) of SNAIL; reduces its transcriptional repressive activity, favors its ubiquitination and degradation and suppresses EMT [145]
			PKA, CK2	Ser11/92	SNAIL stability [157]
			GSK3β	Ser92/96/100/104	SNAIL localization in cytoplasm; β-TRCP-mediated ubiquitination and proteasomal degradation [144,146]
			LATS2	Thr203	increases SNAIL nucleolar retention and activation [148]
			ERK2	Ser82/104	increases SNAIL nuclear accumulation and activation; suppresses E-cadherin; induces EMT [147]
			CK1	Ser104/107	mediates subsequent phosphorylation by GSK3β of S100/96/92, followed by TRCP-mediated ubiquitination and proteasomal degradation
		monoubiquitination	ubiquitin-editing enzyme A2	K296, K234, K235	monoubiquitinated SNAIL has a reduced affinity for GSK3β enzyme; stabilizes in nucleus and escape from degradation by GSK3β-mediated degradation [138]
		O-GlcNAc glycation	Ser112		increases SNAIL stability by suppressing its degradation; disrupts CK1-mediated phosphorylation at S104/107, inhibiting GSK3β-mediated degradation [158]
SLUG/SNAIL2 (encoded by *SNAI2* gene)	has a zinc finger domain with transcriptional repressor activity [138]	phosphorylation	PAK4	Ser158/254	increases transcriptional activity and stabilization as a repressor of the *CDH1* promoter in PC and accelerates cancer progression [159]
GATA3 binding protein 3 (GATA3)	overexpressed in BC; involved in mammary gland development; biomarker for diagnosing BC; tumor suppressor in BC; regulates BC progression depending on cellular context [160]	function modulated by PTMs: acetylation	acetylated by acetyltransferase CBP at K119; deacetylated by HDAC1, HDAC2, HDAC3		inhibits cell migration and invasion in lung adenocarcinoma, downregulating EMT-related transcription factors (SLUG, ZEB1, and ZEB2) [152]
		methylation	Arg261		regulates transactivation of the *Il5* gene in T helper 2 (Th2) cells [161]
		progestin-induces GATA3 phosphorylation	Ser308		induces its proteasome-mediated degradation, decreasing GATA3 levels in BC cells upon PR activation for progestin stimulation of in vitro cell proliferation and in vivo tumor growth [160]
SRY-box transcription factor 2 (SOX2), octamer-binding transcription factor 4 (OCT4), Krűppel-like factor 4 (KLF4), c-MYC	involved in the generation of induced pluripotent stem cells (iPSCs) cells by reprogramming adult cells [153]	acetylation	PKB/AKT, p300		stabilizes KLF4 by inactivating GSK3 [153]

**Table 3:** PTMs of HER2 and EGFR biomarkers in BC.

Classical Biomarkers	Role	PTMs	Modifying Enzymes	PTMs Sites	Effects of PTMs
Human epidermal growth factor receptor 2 (HER2/ErbB2 encoded by *ERBB2* gene)	transmembrane glycoprotein with tyrosine kinase activity, overexpressed in 20–25% of BC [168]	phosphorylation	Y877		in HER2-negative BC cell lines, makes HER2 recognizable by trastuzumab that decreases cell proliferation in HER2-/pHER2+ tumors as in HER2-positive BC cell lines [168,169]
		neddylation	NEDD8 and NAE1		neddylation inhibits HER2 degradation and promotes BC progression; inhibition of neddylation promotes HER2 degradation by improving its ubiquitination [170]
Epidermal growth factor receptor (EGFR/ErbB1/ HER1)	mbEGFR glycoprotein can be internalized in the nucleus of the cell, acting in gene transcription, increasing proliferation, and favoring resistance to chemotherapy [171]; also identified as mtEGFR [27]; half of cases of TNBC and IBC overexpress EGFR that regulates EMT, migration, and invasion [176]	palmitoylation of mtEGFR [27]			promotes mitochondrial fusion and cell survival [27]
		phosphorylation	MEK1/2, ERK1/2	Tyr1068	
		transphosphorylation	Src	Y845 other: Y992/1045/1068/1173	leads to autophosphorylation and kinase activity for Ras/MAPK, PI3/AKT, and JAK-STAT3/5 [177]
				Y1045	regulates EGFR ubiquitination and degradation [177]
		acetylation	CBP acetyltransferase		affects its Tyr phosphorylation, inducing cancer cell resistance to HDAC inhibitors [178]
		ubiquitination and neddylation	c-Cbl ubiquitin ligase, ubiquitin-like molecule NEDD8		induces lysosomal degradation; desensitizes growth-factor-activated receptor tyrosine kinase [179]

**Table 4:** PTMs of cell adhesion and other membrane proteins.

Other Biomarkers	Role	PTMs	Modifying Enzymes	PTMs Sites	Effects of PTMs
Epithelial cell adhesion molecule (EpCAM)	surface protein; biomarker for epithelial tumors and CTCs; upregulated in solid epithelial cancers and stem cells; plays a role in cell adhesion, migration, proliferation, and differentiation [207]	deglycosylation			promotes apoptosis, downregulating Bcl-2 and upregulating Bax and caspase-3 through the ERK1/2 and c-Jun N-terminal kinase mitogen-activated protein kinase signaling pathways [35]
		O-linked glycosylation	Thr171, Thr172		
		N-linked glycosylation	Asn74, Asn111, Asn198		Asn198 is essential for stability maintenance of EpCAM; affects expression level and the molecule lifespan in the plasma membrane [208]
Integrin β1 (ITGβ1)	cell adhesion glycoproteins that connect ECM to the cytoskeleton, involved in cancer progression, cell motility, adhesion, migration, proliferation, differentiation, and chemotherapy resistance [209]	α2,6 sialylation	β-galactosamide α-2,6-sialyltranferase I (ST6Gal-I)		plays critical roles in integrin activation [186]; regulator of cell adhesion and contributes to invasion of BC cells to ECM [195]; integrins are highly α2,6 sialylated on the MDA-MB-231 BC cell line but not on MCF7 BC cells [195]; in human colon tumors, sialylation of ITGβ1 blocks cell adhesion to GAL3 and protects cells against GAL3-induced apoptosis [210]
		N-glycosylation	12 potential N-glycosylation sites, including 7–8, 9–12		ITGβ1 activation positively regulates cell migration, critical for PPIs with other cell membrane proteins (syndecan 4, EGFR) [186]
Integrin β4 (ITGβ4)	transmembrane adhesion molecule; plays a role in invasion, proliferation, EMT, and angiogenesis; putative tumor marker [196]	phosphorylation by EGFR/Src-signaling			enhances malignant potential of BC [72]
		palmitoylation	catalyzed by ZDHHC3		promotes invasive BC cell migration [26]
Scribble (SCRIB)	cell junction and tumor-suppressor protein with a key role in maintaining polarity of epithelial cells; overexpressed and mislocated in human cancers, being involved in the regulation of Hippo pathway in BC cells [173]	palmitoylation			important for SCRIB membrane trafficking, cell polarity, and tumor suppression, emphasizing the importance of palmitoylation for cell polarity and tumorigenesis [173].
Caveolin 1 (CAV1)	membrane-associated scaffolding protein that acts as a tumor suppressor in early stages of cancer and promoter of metastasis; downregulated in human tumors, cancer cell lines and oncogene-transformed cells [211]	phosphorylation by Src tyrosine kinase mediated by RACK1			RACK1 and Src silencing enhances drug sensitivity in MDR cells [74]
Clusterin (CLU1)/apolipoprotein J (APOJ)	glycoprotein/chaperone involved in apoptosis, cell cycle regulation and DNA repair [199]; activity depends on its degree of glycosylation [201]	fucosylation			plays a role in BC progression via proangiogenic cytokines and TNF-α production by intratumoral macrophages [200]
Programmed cell death ligand 1 (PD-L1)	transmembrane protein overexpressed in many cancers, known for its oncogenic function exerted by activation of Wnt/β-catenin pathway [203,204]	palmitoylation			affects its stability; targeting PD-L1 palmitoylation sensitized tumor cells to T-cell killing and inhibited tumor growth [203].

## Data Availability

Not applicable.

## Abbreviations

AKT/PKB—protein kinase B; ARD1—arrest-defective 1 protein; ATM—DNA-sensing kinase ataxia-telangiectasia mutated; AURKA—Aurora A kinase; BC—breast cancer; BMDDCs—bone marrow-derived dendritic cells; β-TRCP—beta transducin repeats-containing proteins; CaMK2—calcium/calmodulin-dependent protein kinase II; CAV1—caveolin 1; CBP—CREB binding protein; CDK4—cyclin-dependent kinase 4; CHIP—carboxyl terminus of Hsc70-interacting protein; CK2-casein kinase IICul3^SPOP^-cullin 3–SPOP ubiquitin E3 ligase; CTCs—circulating tumor cells; EC—endometrial cancer; ERK1/2—extracellular-signaling-regulated kinase 1/2; FAK—focal adhesion kinase; GAL3—galectin 3; GSK3β—glycogen synthase kinase 3β; GSTP—glutathione S-transferase Pi; HBO1—histone acetyltransferase binding to ORC1; HDAC—histone deacetylases; IBC—inflammatory breast cancer; IKKα—inhibitory-κB kinase; ITGβ4—integrin β4; JNK—c-Jun terminal kinases; KATs—lysine acetyltransferases; LNCaP—androgen-sensitive human prostate adenocarcinoma cells; MAPKs—p38 mitogen-activated protein kinases; MDM2—mouse double minute 2 homolog/E3 ubiquitin-protein ligase; MDR—multidrug resistant; MMP—metalloproteinase; MOF—males-absent on the first; mtEGFR—mitochondrial epidermal growth factor receptor; NAE1—NEDD8-activating enzyme E1 subunit 1; NO—nitric oxide signaling pathway; OC—ovarian cancer; PAK4—p21-activated kinase; PAK1—p21-activated kinase 1; PC—prostate cancer; PCAF—P300/CBP-associated factor; PDAC—pancreatic ductal adenocarcinoma; PKA—protein kinase A; PKC—protein kinase C; PKD1—protein kinase 1; PRMT1—protein arginine methyltransferase 1; RACK1—receptor for activated C kinase 1; SENP1—SUMO-specific peptidase 1; SUMO1—small ubiquitin-like modifier; TKs—tyrosine kinases; TYR—tyrosine; ULK1—Unc-51-like kinase 1; UPS—ubiquitin proteasome system; USP—ubiquitin-specific protease/peptidase; ZDHHC—zinc finger DHHC-type palmitoyl acyltransferase.
